# Germline BRCA testing in Denmark following invasive breast cancer: Progress since 2000

**DOI:** 10.2340/1651-226X.2025.42418

**Published:** 2025-01-28

**Authors:** Aleksandar M. Kostov, Maj-Britt Jensen, Bent Ejlertsen, Mads Thomassen, Maria Rossing, Inge S. Pedersen, Annabeth H. Petersen, Lise Lotte Christensen, Karin A.W. Wadt, Anne-Vibeke Lænkholm

**Affiliations:** a Department of Surgical Pathology, Zealand University Hospital, Roskilde, Denmark; b Danish Breast Cancer Group, Department of Oncology, Copenhagen University Hospital, Rigshospitalet, Denmark; c Department of Clinical Medicine, Faculty of Health and Medical Sciences, University of Copenhagen, Denmark; d Department of Clinical Genetics, Odense University Hospital, Odense, Denmark; e Human Genetics, Department of Clinical Research, University of Southern Denmark, Odense, Denmark; f Clinical Genome Center, University of Southern Denmark and Region of Southern Denmark, Odense, Denmark; g Department of Genomic Medicine, Copenhagen University Hospital, Rigshospitalet, Copenhagen, Denmark; h Department of Clinical Medicine, Aalborg University, Aalborg, Denmark; i Department of Molecular Diagnostics, Aalborg University Hospital, Aalborg, Denmark; j Clinical Cancer Research Center, Aalborg University Hospital, Aalborg, Denmark; k Department of Clinical Genetics, Vejle Hospital, University Hospital of Southern Denmark, Denmark; l Department of Molecular Medicine (MOMA), Aarhus University Hospital, Aarhus, Denmark; m Department of Clinical Genetics, Copenhagen University Hospital, Rigshospitalet, Copenhagen, Denmark

**Keywords:** Genetic testing, hereditary breast cancer, pathogenic variants, Danish Breast Cancer Group

## Abstract

**Background and purpose:**

Despite advancements in genetic testing and expanded eligibility criteria, underutilisation of germline testing for pathogenic variants in *BRCA1* and *BRCA2* (*BRCA*) remains evident among breast cancer (BC) patients. This observational cohort study presents real-world data on *BRCA* testing within the context of clinical practice challenges, including incomplete family history and under-referral.

**Material and methods:**

From the Danish Breast Cancer Group (DBCG) clinical database, we included 65,117 females with unilateral stage I–III BC diagnosed in 2000–2017, of whom 9,125 (14%) were *BRCA* tested. Test results spanned from 1999 to 2021. We evaluated test rates overall and in three diagnosis periods. In logistic regression models, we examined the correlation between a *BRCA* test and patients’ age, residency region, receptor status, and diagnosis period.

**Results:**

Test rates rose most significantly among patients aged under 40 years, increasing from 47% (2000–2005) to 88% (2012–2017), albeit with regional discrepancies. Test timing shifted in recent years, with most results within 6 months of BC diagnosis, primarily among the youngest patients. *BRCA* test rates were higher for oestrogen receptor-negative/human epidermal growth factor receptor 2-negative BC (25% in 2000–2005 vs. 38% in 2012–2017), and these findings were confirmed in multivariate regression models.

**Interpretation:**

Our results indicate a critical need for an intensified focus on *BRCA* testing among BC patients older than 40, where a mainstreamed testing approach might overcome delayed or missed testing. Current DBCG guidelines recommend *BRCA* testing of all BC patients younger than 50 years, while a general recommendation for older patients is still missing.

## Introduction

Germline testing of the *BRCA1* and *BRCA2* genes (*BRCA*) has been well-established for more than two decades for women with invasive breast cancer (BC) [1]. Rapid progress in next-generation sequencing (NGS) has made genetic testing faster and more cost-efficient and broadened the criteria for germline testing, thereby increasing the number of eligible patients [2–4]. Patients with pathogenic variants (PV) in *BRCA* (*BRCA* carriers) often present at a younger age than non-*BRCA* carriers and have an increased risk of contralateral BC and ovarian cancer (OC) [5]. Consequently, *BRCA* carriers are offered risk-managing programs with intensified follow-up and risk-reducing surgery (contralateral mastectomy alone or in sequence with bilateral salpingo-oophorectomy) [1, 5, 6]. Current recommendations for genetic testing from the Danish Breast Cancer Group (DBCG) are consistent with national guidelines across Europe and focus on the family predisposition, early age onset of BC (under 50 years), bilateral BC, Triple Negative (TNBC), that is, oestrogen receptor-negative (ER-), human epidermal growth factor receptor 2 (HER2)-negative, progesterone receptor (PR)-negative and/or basal-like molecular subtype, BC and OC in the same patient and male BC [4, 7]. Recently, several international guidelines have broadened the inclusion criteria, recommending *BRCA* testing to all newly diagnosed BC patients when 65 years or younger, to candidates for poly(ADP-ribose) polymerase inhibitor (PARPi) therapy, and patients with TNBC [8–10]. Similarly, testing women under the age of 60 with BC is recommended in Norway based on a 90% sensitivity for *BRCA* PV [11, 12].

Underdiagnosis of *BRCA* among patients with BC has been in focus in recent years, suggesting that up to half of *BRCA* carriers do not meet current guidelines and mainstreamed genetic testing is highly recommended [13–16]. In other Nordic countries, recent studies emphasised the risk of missing, especially *BRCA2* carriers, and showed unequal *BRCA* testing rates, which differed among all patient groups and geography [11, 17]. Previous reporting on *BRCA* testing rates has been based on patient questionnaires or cross-sectional study estimates, and the effectiveness of testing criteria is still unclear [16, 18, 19]. In the context of the clinical day-to-day practice challenges, including incomplete family history and under-referral, documenting real-world data on *BRCA* testing becomes particularly relevant.

In this nationwide observational cohort study, we aimed to describe *BRCA* testing trends among stage I–III unilateral BC patients in the five Danish regions from 2000 to 2017, based on the hypothesis of increased accessibility for *BRCA* testing over time. Specifically, we aimed to assess testing rates in patients under 40 and those with TNBC, aligning with Danish guidelines in the study period.

## Patients and methods

### Cohort

**Figure 1:**
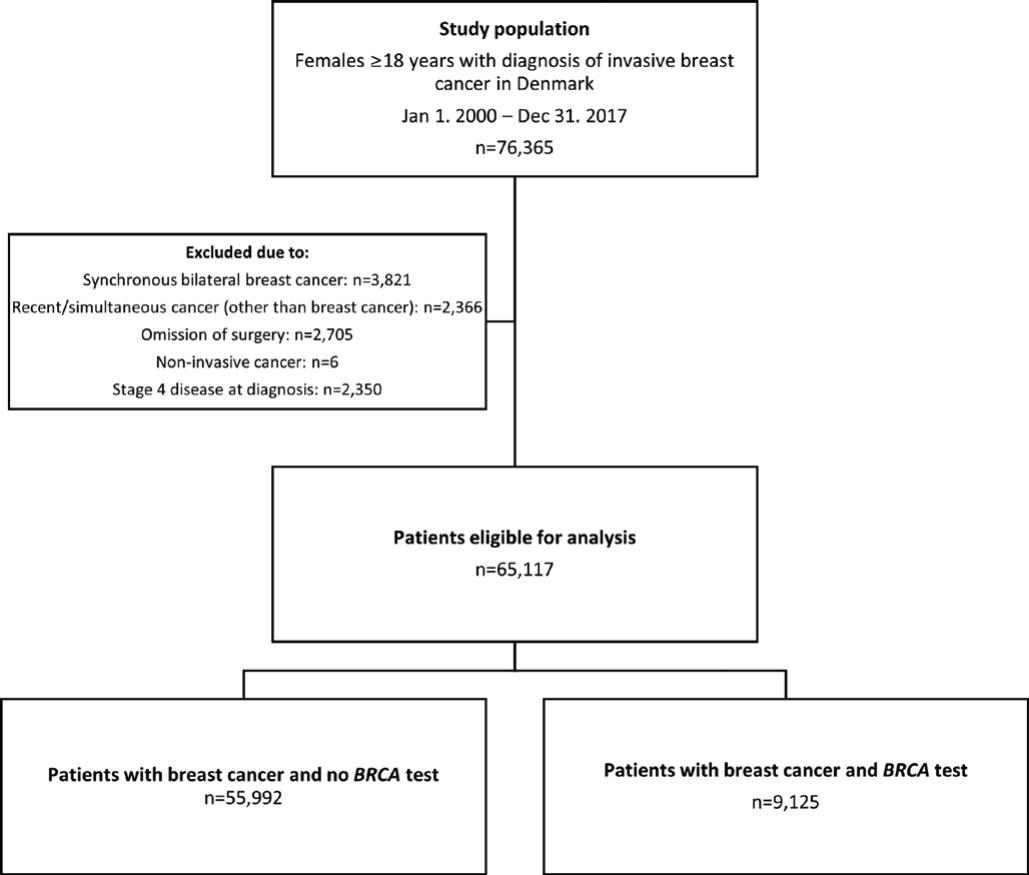
Trial profile.

From the clinical database of the DBCG, we gathered a cohort of 76,365 females aged 18 years or older with a biopsy or primary surgical specimen with BC between Jan 1, 2000, and Dec 31, 2017. We linked primary data with data from the Danish Pathology Data Bank, the Danish Cancer Registry, and the Danish National Patient Registry using the central personal register number, a unique identifier assigned to all Danish residents at birth or immigration [20–23]. We excluded 11,248 patients (Figure 1): 3,821 due to synchronous bilateral BC, 2,350 due to stage IV disease at or within 90 days after diagnosis, 2,705 due to the omission of surgery, six due to non-invasive disease, and 2,366 due to previous or simultaneous invasive cancer (other than BC) within 200 days after diagnosis. Long-term survivors, surpassing 9.5 years from another invasive cancer diagnosis, were not excluded. The study complies with the STROBE guidelines for reporting [24].

### Genetic test results

*BRCA* testing was implemented for diagnostic purposes in 1997 at Rigshospitalet, Copenhagen University Hospital, and in 1999 at a national level [25]. The five laboratories (Dept. of Genomic Medicine, Copenhagen University Hospital, Rigshospitalet; Dept. of Clinical Genetics, Odense University Hospital; Dept. of Molecular Diagnostics, Aalborg University Hospital, Dept. of Clinical Genetics, Vejle Hospital; and Dept. of Molecular Medicine (MOMA), Aarhus University Hospital) performed *BRCA* testing. Since 2011, the analytic platform was NGS gene panel testing, replacing Sanger sequencing. A detailed description of the Danish national concerted efforts of *BRCA* classification has been published previously [25]. *BRCA* test results, including the requisition date, were uploaded to the DBCG repository.

Missing data on *BRCA* test results was retrieved from the five laboratories before the statistical analysis. The oldest result was maintained in the case of multiple testing (e.g., screening test after a previous predictive test result). New results were only considered in the case of variant reclassification. The patients in this study were tested from Sep 1999 to Dec 2021.

### Statistical analysis

All statistical analyses were conducted in R for Windows, version 4.3.2. We have summarised data using frequency counts and percentages in Table 1. The median age was calculated together with the interquartile range (IQR). ER and HER2 were analysed together since guidelines focus on TNBC. PR is not scored routinely in Denmark; hence, TNBC was defined as ER-negative/HER2-negative.

**Table 1:** Characteristics of the study population, overall, and for patients with a *BRCA* test (w/test).

	Total			Year of diagnosis								
				2000–2005			2006–2011			2012–2017		
	No.	w/Test	(%)	No.	w/Test	(%)	No.	w/Test	(%)	No.	w/Test	(%)
Overall	65117	9125	(14)	18682	1891	(10)	23405	3122	(13)	23030	4112	(18)
Region												
North	5907	699	(12)	1479	140	(9)	2237	263	(12)	2191	296	(14)
Central	12668	1630	(13)	3257	294	(9)	4694	602	(13)	4717	734	(16)
Southern	15747	2426	(15)	4948	566	(11)	5604	817	(15)	5195	1043	(20)
Capital	20937	3040	(15)	6277	600	(10)	7195	989	(14)	7465	1451	(19)
Zealand	9858	1330	(13)	2721	291	(11)	3675	451	(12)	3462	588	(17)
Age at diagnosis												
18–39	2827	2043	(72)	873	409	(47)	937	744	(79)	1017	890	(88)
40–59	26224	5414	(21)	8197	1237	(15)	9142	1759	(19)	8885	2418	(27)
≥ 60	36066	1668	(5)	9612	245	(3)	13326	619	(5)	13128	804	(6)
Histologic type												
Ductal	52125	7601	(15)	15042	1563	(10)	18900	2647	(14)	18183	3391	(19)
Lobular	7059	753	(11)	2168	185	(9)	2317	232	(10)	2574	336	(13)
Other or unknown	5933	771	(13)	1472	143	(10)	2188	243	(11)	2273	385	(17)
ER/HER2 ^1^												
ER-/HER2-	5281	1600	(30)	915	229	(25)	2170	538	(25)	2196	833	(38)
ER-/HER2+	2638	527	(20)	629	123	(20)	1178	212	(18)	831	192	(23)
ER-/ HER2 Unknown	2611	161	(6)	2199	126	(6)	375	31	(8)	37	4	(11)
ER+/HER2-	36254	5135	(14)	2602	731	(28)	15311	1880	(12)	17341	2524	(15)
ER+/HER2+	4894	1088	(23)	701	219	(31)	1905	337	(18)	2288	532	(23)
ER+/HER2 Unknown	13855	604	(4)	11420	460	(4)	2257	120	(5)	178	24	(13)
ER Unknown	584	10	(2)	216	3	(1)	209	4	(2)	159	3	(2)
Malign. grade												
Grade I	16966	1751	(10)	4846	436	(9)	6718	694	(10)	5402	621	(11)
Grade II	26380	3481	(13)	7266	695	(10)	9205	1187	(13)	9909	1599	(16)
Grade III	13787	2789	(20)	3948	524	(13)	4801	911	(19)	5038	1354	(27)
Not graded	7984	1104	(14)	2622	236	(9)	2681	330	(12)	2681	538	(20)
Nodal status^2^												
Negative	36494	5045	(14)	9104	950	(10)	12722	1616	(13)	14668	2479	(17)
1–3 positive LN	17751	2581	(15)	5552	603	(11)	6849	1008	(15)	5350	970	(18)
≥ 4 positive LN	8157	1091	(13)	3282	332	(10)	3014	415	(14)	1861	344	(18)
FNA positive	1151	361	(31)	12	2	(17)	348	68	(20)	791	291	(37)
Unknown	1564	47	(3)	732	4	(1)	472	15	(3)	360	28	(8)
Tumour size^3^												
0–10 mm	12824	1681	(13)	2763	305	(11)	4809	643	(13)	5252	733	(14)
11–20 mm	27004	3824	(14)	7590	845	(11)	9678	1301	(13)	9736	1678	(17)
21–50 mm	22416	3168	(14)	7405	660	(9)	7920	1044	(13)	7091	1464	(21)
>50 mm	2368	399	(17)	774	69	(9)	794	118	(15)	800	212	(27)
Unknown	505	53	(10)	150	12	(8)	204	16	(8)	151	25	(17)
Comorbidity^4^												
CCI 0	52064	8046	(15)	15255	1725	(11)	18695	2747	(15)	18114	3574	(20)
CCI 1	8105	755	(9)	2107	119	(6)	2936	264	(9)	3062	372	(12)
CCI ≥ 2	4948	324	(7)	1320	47	(4)	1774	111	(6)	1854	166	(9)

1 HER2- = HER2-negative, HER2+ = HER2-positive;

2 Nodal status applies to clinical node status (radiologically determined with ultrasound or MRI) or fine-needle aspirate (FNA) from axillary nodes for patients referred to neoadjuvant chemotherapy. Otherwise, the pathological nodal status was applied. If axillary dissection was performed and no lymph nodes (LNs) were found, the nodal status was determined as ‘Unknown’;

3 For patients who had received neoadjuvant chemotherapy, the clinical tumour size (radiologically determined by ultrasound or MRI) was applied. For patients operated upfront, the pathological tumour size was applied;

4 Charlson’s Comorbidity Index (CCI) was calculated using ICD-8 and ICD-10 codes for 19 chronic diseases recorded during hospital contacts in the 10 years before the BC diagnosis and were retrieved from the Danish National Patient Registry.

Univariate and multivariate logistic regression (LR) models with the binary outcome *BRCA* test (yes vs. no) were applied to present odds ratios (ORs) according to age and year at diagnosis, region of residency, and receptor status. Other tumour characteristics not directly referred to in the guidelines for genetic testing were not included in the models. We evaluated the interaction terms between age at diagnosis and year of diagnosis, and receptor status and year of diagnosis. Overall *p*-values were derived from Likelihood-ratio tests, where a *p*-value of less than 0.05 was considered significant.

We conducted Hosmer and Lemeshow goodness-of-fit tests. In all tests, the *p*-values were below 0.05, indicating a poor fit, except for the multivariate model, including interaction terms of age and year of diagnosis, with a *p*-value of 0.13, suggesting a good predictive model for *BRCA* testing.

## Results

### Study cohort

Between Jan 1, 2000 and Dec 31, 2017, 76,365 women were diagnosed with BC in Denmark, and 65,117 (85%) were eligible for the study (Figure 1). Median age at diagnosis was 61 years (IQR 52–69), and the majority (80%) of patients (Table 1) were non-comorbid and diagnosed with invasive ductal carcinoma of the breast with ER+/HER2-negative status (56%). Tumours were most frequently malignancy grade II (41%). Forty-two per cent of patients had node-positive disease. Most tumours measured between 11 and 20 mm (41%).

### Testing distribution

One or more tests were linked to 9,125 (14%) patients (Table 1). Test rates increased from 10% of patients diagnosed in 2000–2005 to 18% of patients diagnosed in 2012–2017. The median age was 48 years (40–56). Among patients who were 18–39 years (4% of the cohort) at diagnosis, 72% were tested compared to 21% of those aged 40–59 years (40%) and 5% of those 60 years or older (55%) (Table 1).

Overall, 30% of patients with TNBC had a *BRCA* test, with test rates increasing from 25% in the first period to 38% in the final period. *BRCA* test rates were higher in HER2-positive BC (20–23%) compared to 14% in the most frequent receptor combination, ER+/HER2-normal. In the case of unknown receptor status, test rates were low. However, the proportion of patients with unknown HER2 status decreased drastically after 2006. We observed increasing test rates with higher malignancy grade and larger tumour size and decreasing test rates with increasing comorbidity index. Test rates of around 14% were observed across groups of nodal status. Across the five Danish regions, test rates were around 10% initially. In the final period, test rates increased to 14–20%, with regional differences widening, as shown in Table 1.

### Test timing

Among the 9,125 women with a *BRCA* test, only 173 (2%) women were tested before BC diagnosis, while 7,145 (78%) were tested at or after BC diagnosis and before a second event, defined as any BC recurrence, other malignancy, or death. Lastly, 1,807 (20%) were tested after a second event. Among the 7,145 tested before a second event, test results were available for 2,169 (30%) available within 6 months, 1,663 (18%) after 6 to less than 12 months, 997 patients (11%) between the first and second year, and 2,316 (25%) more than 2 years after diagnosis. Among these 7,145, 720 (10%) had a confirmed *BRCA* PV: 267 (15%) of patients aged 18–39, 378 (12%) of those aged 40–59, and 75 (7%) of those aged 60 and older.

**Figure 2:**
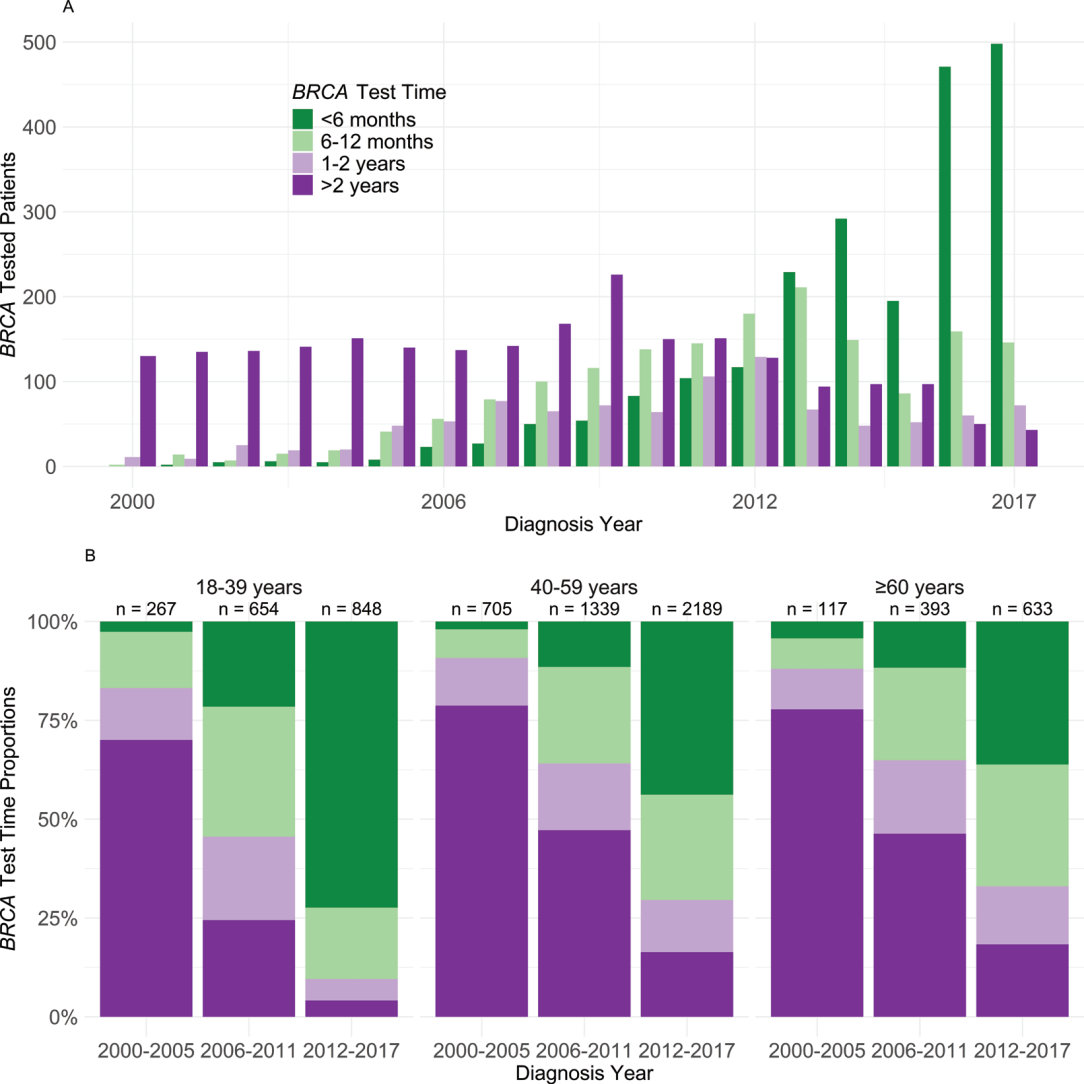
(a) *BRCA* test timing according to year of BC diagnosis among 7,145 patients with *BRCA* test after unilateral stage I–III BC and before a second event. (b) Distribution of *BRCA* test timepoint by age and year of diagnosis, among 7,145 patients with *BRCA* test after unilateral stage I–III BC and before a second event.

The number of patients with available results within 6 months after diagnosis, reflecting timely testing, rose from zero in 2000 to 498 in 2017 (Figure 2A). More than 70% of patients aged under 40 and diagnosed between 2012 and 2017 received timely testing, compared to less than half of those aged 40 and older (Figure 2B).

### Logistic regression models

In univariate LR models, we found a significant correlation between patients referred for *BRCA* testing and: age at diagnosis, region of residency, year of diagnosis, and receptor status (all *p*-values < 0.0001). Table 2 shows OR for patients having a *BRCA* test, where OR > 1 favoured *BRCA* testing. Testing probability was significantly lower (*p* < 0.0001) for patients aged 40–59 years (OR = 0.10 [0.09–0.11]) and 60 years or older (OR = 0.02 [0.02–0.02]) compared to the reference age group 18–39. ORs for *BRCA* testing nearly doubled for the diagnosis period 2012–2017 (OR = 1.93 [1.83–2.05]) compared to the reference period 2000–2005. Residency in other regions besides Southern Denmark and the Capital region showed a significantly lower probability of *BRCA* testing (*p* < 0.0001). Compared to TNBC, other receptor combinations showed lower testing probability (*p* < 0.0001).

**Table 2:** Logistic regression models, where Odds ratio > 1.0 favours *BRCA* testing.

	Univariate			Multivariate		
	Odds ratio	[95% CI]	*P*	Odds ratio	[95% CI]	*P*
Age			< 0.0001			< 0.0001
18–39	Ref			Ref		
40–59	0.10	[0.09–0.11]		0.10	[0.10–0.11]	
≥ 60	0.02	[0.02–0.02]		0.02	[0.02–0.02]	
Year of diagnosis			< 0.0001			< 0.0001
2000–2005	Ref			Ref		
2006–2011	1.37	[1.29–1.45]		0.87	[0.80–0.94]	
2012–2017	1.93	[1.82–2.05]		1.22	[1.13–1.33]	
Region			< 0.0001			< 0.0001
Capital	Ref			Ref		
Central	0.87	[0.81–0.93]		0.87	[0.81–0.93]	
North	0.79	[0.72–0.86]		0.81	[0.74–0.90]	
Zealand	0.92	[0.86–0.98]		0.94	[0.87–1.02]	
South	1.07	[1.01–1.14]		1.08	[1.01–1.15]	
ER/HER2			< 0.0001			< 0.0001
ER-/HER2-	Ref			Ref		
ER-/HER2+	0.57	[0.51–0.64]		0.54	[0.47–0.61]	
ER+/HER2-	0.39	[0.37–0.42]		0.50	[0.46–0.54]	
ER+/HER2+	0.66	[0.60–0.72]		0.60	[0.54–0.66]	
Unknown	0.11	[0.10–0.12]		0.14	[0.13–0.16]	

CI: confidence interval.

Similar estimates for *BRCA* testing probabilities were estimated in the multivariate LR-model. However, the increased ORs for testing in later periods, compared to 2000–2005, were highly modulated by the receptor status, correlated with the decrease of HER2 unknown from 67% in the first period to 1% in the last (Table 1). In the multivariate LR model (Table 2), the ORs dropped to 0.87 [0.80–0.94] in the second diagnosis period, while OR increased again in the last diagnosis period (1.22 [1.13–1.33]).

In interaction tests, we explored *BRCA* testing probabilities according to age at diagnosis and receptor status over time (Table 3). There was strong evidence for interactions (*p* < 0.0001), showing that increasing testing odds over time was primarily driven by increased testing in the youngest age group and in patients with TNBC (OR = 4.78 and OR = 3.63, respectively), while testing odds did not increase significantly for patients over 40 at diagnosis.

**Table 3:** Multivariate logistic regression models with interactions, where Odds ratio>1.0 favours BRCA testing.

Diagnosis year	2000–2005	2006–2011		2012–2017		*P*
Age at diagnosis^1^		Odds ratio	[95% CI]	Odds ratio	[95% CI]	< 0.0001
18–39	Ref	2.75	[2.21–3.41]	4.78	[3.77–6.07]	
40–59	Ref	0.71	[0.65–0.78]	1.06	[0.97–1.16]	
≥ 60	Ref	0.79	[0.68–0.93]	0.96	[0.82–1.13]	
ER/HER2^2^		Odds ratio	[95% CI]	Odds ratio	[95% CI]	< 0.0001
ER-/HER2-	Ref	1.48	[1.22–1.78]	3.63	[3.00–4.38]	
ER-/HER2+	Ref	1.28	[0.99–1.67]	1.83	[1.38–2.45]	
ER+/HER2-	Ref	0.71	[0.71–0.97]	0.87	[0.80–0.96]	
ER+/HER2+	Ref	0.93	[0.81–1.21]	1.25	[1.03–1.54]	
Unknown	Ref	2.16	[1.75–2.66]	1.79	[1.05–3.05]	

CI: confidence interval.

1 Odds ratios for the interaction term of age at diagnosis and diagnosis year in multivariate LR model, adjusted for region of residency and receptor status;

2 Odds ratios for the interaction term of receptor status and diagnosis year in the multivariate LR model, adjusted for region of residency and age at diagnosis.

HER2-positive status was related to increasing *BRCA* testing odds only in the most recent period, irrespective of the ER status. The frequent receptor combination (ER+/HER2-negative) did not correlate with increasing odds for *BRCA* testing. Table 4 shows test rates for age and receptor status combination for the first and latest time periods. Test rates increased over time for patients under 40 years, regardless of their receptor status and for TNBC across all age groups.

**Table 4:** Incidence of BC (No.) in 2000–2005 and 2012–2017 by age at diagnosis corresponding proportions of *BRCA* tested patients (% Test) according to tumour receptor status.

Age at diagnosis	2000–2005^1^						2012–2017^2^					
	18–39		40–59		≥ 60		18–39		40–59		≥ 60	
	No.	% Test	No.	% Test	No.	% Test	No.	% Test	No.	% Test	No.	% Test
ER/HER2												
ER-/HER2-	122	63	505	25	288	9	254	92	848	52	1094	15
ER-/HER2+	69	57	331	21	229	6	72	89	357	29	402	6
ER+/HER2-	218	67	1608	32	776	9	501	86	6530	24	10310	5
ER+/HER2+	114	61	415	33	172	6	177	88	1038	31	1073	5
Unknown	350	22	5338	7	8147	1	13	62	112	15	249	2

1 No. = 18,682,

2 No. = 23,030.

## Discussion

Among 65,117 Danish females diagnosed with unilateral early BC between 2000 and 2017, 14% had undergone *BRCA* testing between 1999 and 2021. Test rates increased from 10% in 2000–2005 to 18% in 2012–2017, with the most prominent increase observed in females younger than 40 at BC (from 50 to 88% in the same periods). This increase reflects the recommendations for testing BC patients aged 40 or younger in the last diagnosis period, and it was higher than the 66% test rate reported among this patient group in Georgia and California from 2013 to 2017 [18]. An even lower test rate of 23% was reported in the 2015 Cancer Control Module of the US National Health Interview Survey among BC patients diagnosed before age 45 [19]. However, the results were based on self-reported survey answers from a small sample size (*n* = 325) with a significant risk of recall bias [19].

Despite the significant increase in testing among younger patients, the rise was less prominent among older patients, leading to lower overall test rates. Consequently, less than 20% of Danish BC patients were tested in the most recent period, revealing lower rates compared to other countries [11, 18, 19]. In Norway, 35% of women diagnosed with BC in 2016 and 2017 were tested, corresponding to 75% of patients who met the national test guidelines [11]. The high test rates in Norway were attributed to the direct offering of the *BRCA* test to patients by surgeons or oncologists, a practice known as mainstreaming [11]. The incidence of bilateral BC in the Norwegian study was 1%, but the cases were not specified as synchronous [11]. In our study, this rate was 5%, and including these patients still would not match test rates in Norway. *BRCA* testing rates were highest in the Capital and Southern Regions hosting the University Hospitals in Copenhagen, Rigshospitalet, and Odense. Limited focus on *BRCA* testing and longer travel distances may explain lower test rates in smaller centres. Additionally, Region Zealand did not offer oncogenetic counselling before 2017, where patients were mainly referred to Rigshospitalet. Several Danish centres currently provide genetic counselling via video after local blood sample collection to accommodate long-distance patients. Implementing the Norwegian or similar guidelines in Denmark will likely increase *BRCA* testing and associated costs. The Danish healthcare system is publicly financed and requires all recommendations to be accepted by the Danish Regions and included in their economic frameworks [26]. Since 2013, multigene-panel testing has been implemented universally in Denmark and will increase the variants of unknown significance (VUS) and PV rates in moderate-risk genes [18, 25, 27]. By implementing mainstreamed *BRCA* testing, surgeons and oncologists could share normal results, referring only patients with PV or VUS who require genetic counselling expertise.

A cost-effective population-based test for three founder mutations has since 1996 in Poland resulted in *BRCA* testing of 500,000 people, but the study did not report specific test rates among patients with BC [28]. The absence of widespread founder mutations prohibits a similar strategy from being successful in Denmark [29].

We observed a significant shift in test timing as none of the patients in 2000 received their test results within 6 months, while most patients (65%) diagnosed in the last two study years received timely *BRCA* results. Similar *BRCA* testing time trends have been observed by others [18]. Despite the well-established predictive *BRCA* testing in hereditary breast and ovarian cancer syndrome (HBOC) families in Denmark, evidence shows that very few individuals are linked to an HBOC family [25, 29]. This fact is underscored by the low number of patients in our cohort who had undergone *BRCA* testing before a BC diagnosis.

In our study, patients with TNBC were tested more frequently than those with other receptor subtypes (30% vs. 2–23%), with test rates increasing from 25% in 2000–2005 to 38% in 2012–2017. The increase is likely due to the 2016 DBCG guidelines recommending genetic testing for TNBC patients under 60 and might be most pronounced in the last study year [30]. Multivariable LR models suggested a similar correlation, showing an increased probability for *BRCA* testing in patients with TNBC diagnosed in 2006–2011, likely reflecting awareness of the higher prevalence of TNBC among *BRCA* carriers [31]. We also observed increased *BRCA* testing for HER2-positive BC, although not as high as for TNBC. However, these correlations seem highly modulated by, first, changed national diagnostic practices, with HER2 routinely scored since 2006 and second, the analysis of ER unknown and HER2 unknown as one group in the LR models. ER unknown proportions were constant over time, while HER2 unknown decreased rapidly. Unlike other known ER and HER2 combinations, where the incidence doubled after routinely scoring HER2, the incidence of patients with the most common receptor combination, ER+/HER2-negative, increased sixfold after 2005 (Table 1). Consequently, the observed increase in the TNBC and HER2+ groups might also apply to the ER+/HER2- group. In this case, the effect of the increased number of patients with *BRCA* tests, as shown in Table 1, appears diluted and may be falsely reflected as higher odds for the ER/HER2 unknown group. Despite HER2-positive BC occurring less frequently among *BRCA* carriers, studies have indicated that patients with this combination have poorer prognoses [32, 33]. Our findings highlight the importance of *BRCA* testing in patients with other receptor subtypes than TNBC, given its potential impact on treatment decisions and patient outcomes. Even though DBCG guidelines recommend PARPi to selected *BRCA*-mutated patients who meet the OlympiA trial inclusion criteria, PARPi is not currently approved in Denmark for adjuvant treatment of early BC [34].

Our study is the first to evaluate *BRCA* test trends in Denmark. A key strength is reporting real-world data on *BRCA* testing among over 65,000 BC patients from all testing sites during three periods of 6 years each, unlike previous cross-sectional estimates or single-site studies [16, 18, 19]. Furthermore, the high completeness of centralised data, the integration of several Danish registries, and the collaboration of the genetic laboratories to provide updated *BRCA* results have ensured data robustness. In multivariate LR models with interaction terms, we identified factors that decreased the probability of *BRCA* testing for patients with unilateral BC, for whom guidelines might be insufficient. The predefined test timing intervals (i.e., *BRCA* test within 6 months, 6–12 months, 1–2 years and later than 2 years after BC) were based on similar intervals as previously described [18]; however, the DBCG guidelines do not state when the *BRCA* test should be done. Due to unavailable data, we could not account for patients tested based on pedigree or those who rejected genetic testing. Even though our inclusion period spans over 18 years, our results might not reflect the broadening of the test criteria to all patients younger than 50 at BC and all TNBC patients in 2022. However, extending the inclusion period by an additional 6 years would hinder assessing patients who undergo late testing (more than 2 years after BC).

## Conclusion

The rise in *BRCA* testing of BC patients in Denmark reflects an increased awareness and accessibility due to cheaper NGS testing. Still, test rates increased slower than in other countries, especially for patients older than 40 and for the most common receptor combination, that is, ER-positive/HER2-negative [11, 18]. Current DBCG guidelines recommend *BRCA* testing for all patients under 50 at BC, while international guidelines recommend testing patients up to age 65, leaving a large gap [8–10]. Implementing mainstreamed genetic testing of patients with surgically treatable BC could eliminate the geographical discrepancy promoting timely detection of *BRCA* PV and a risk-reducing approach, also beyond young age and TNBC.

## Data Availability

The findings of this study are supported by data which can be provided upon request and approval from the DBCG. Due to institutional restrictions and restrictions by the Danish Health Act, data may not be publicly available.
